# Supplementary material for: First Immunoassay for Measuring Isoaspartate in Human Serum Albumin
Source: Molecules. 2021 Nov 5;26(21):6709. doi: 10.3390/molecules26216709 (PMC8587401; doi:10.3390/molecules26216709)
Supplement: Supplementary file 1 [file molecules-26-06709-s001.zip › molecules-1392177-supplementary.pdf]

Supplementary materials

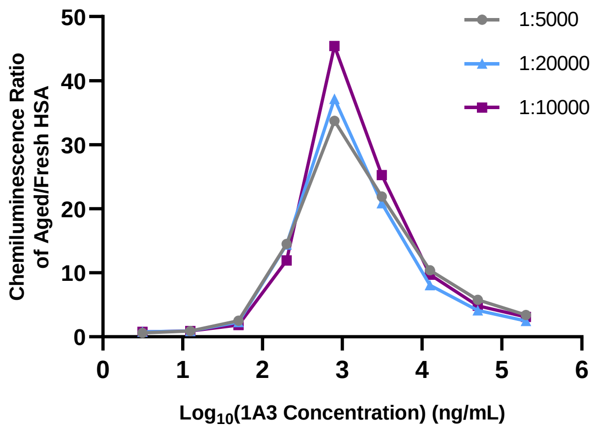

Figure S1. Criss-cross ELISA results illustrated that the specificity of 1A3 reaches highest when primary 1A3 antibody is 800 ng/mL and the dilution ratio of the secondary antibody is 1:10000.

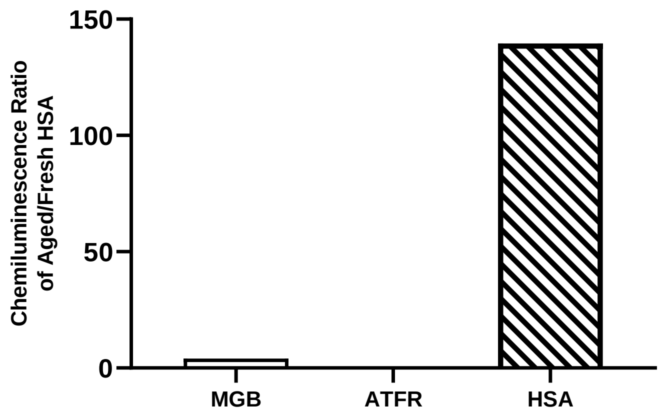

Figure S2. 1A3 has no specificity towards aged human transferrin (TFR) or myoglobin from equine skeletal muscle (MGB) that were artificially deamidated for 7 days.

Table S1. Information of plasma samples from 100 healthy donors. All tested negatively to human immunodeficiency virus (HIV), hepatitis B virus surface antigen (HBsAg) and hepatitis C virus (HCV).

Sample No.	Gender	Age, years	Sample No.	Gender	Age, years
1	F	37	51	M	48
2	F	37	52	M	37
3	F	27	53	M	21
4	F	55	54	M	29
5	F	29	55	M	40
6	F	41	56	M	36
7	F	39	57	M	28
8	F	43	58	M	35
9	F	63	59	M	29
10	F	20	60	M	31
11	F	22	61	M	30
12	F	26	62	M	53
13	F	31	63	M	22
14	F	33	64	M	38
15	F	45	65	M	46
16	F	24	66	M	27
17	F	58	67	M	40
18	F	62	68	M	53
19	F	25	69	M	28
20	F	29	70	M	35
21	F	41	71	M	54
22	F	26	72	M	25
23	F	57	73	M	59
24	F	39	74	M	28
25	F	65	75	M	48
26	F	29	76	F	40
27	M	44	77	M	20
28	M	48	78	F	37
29	M	35	79	M	25
30	M	35	80	F	48
31	M	29	81	M	35
32	M	33	82	F	32

33	M	27	83	F	25
34	M	22	84	M	27
35	M	28	85	F	25
36	M	31	86	M	33
37	F	21	87	M	44
38	F	48	88	M	26
39	F	37	89	M	35
40	M	33	90	M	33
41	M	59	91	M	26
42	M	30	92	F	26
43	F	37	93	M	24
44	M	44	94	F	59
45	M	30	95	F	25
46	M	54	96	M	32
47	M	38	97	F	23
48	M	45	98	M	29
49	M	26	99	M	25
50	M	34	100	F	35
